# SCP-DETR: A efficient small-object-enhanced feature pyramid approach for PCB defect detection

**DOI:** 10.1371/journal.pone.0330039

**Published:** 2025-08-29

**Authors:** Yuanyuan Wang, Tongtong Yin, Xiuchuan Chen, Yemeng Zhu, Jibin Wang, Yonghao Ma, Luyue Liu, Jiajun Wang

**Affiliations:** 1 College of Computer and Software Engineering, Huaiyin Institute of Technology, Huaian, China; 2 Nanjing Howso Technology Co., Ltd, Nanjing, China; Institut Sains dan Teknologi Terpadu Surabaya, INDONESIA

## Abstract

Defects generated during PCB manufacturing, transportation, and storage seriously impact the quality and performance of electronic components. However, detection accuracy is limited due to excessive background interference and the small size of defect targets. To alleviate these issues, this paper proposes an improved PCB defect detection method based on RT-DETR, named SCP-DETR. Firstly, to effectively detect small targets, the S2 feature layer is incorporated into the neck feature fusion. While this improves detection capability, it also introduces considerable computational overhead. To mitigate this, we use SPDConv (Space-to-Depth Convolution) to process the S2 feature layer, reducing the computational complexity. The processed S2 feature layer is then fused with the S3 feature layer and higher-level features. Subsequently, we feed these features into a specially designed CO-Fusion module. By embedding our proposed CSPOKM(CSP Omni-Kernel Module) into the original fusion module, the CO-Fusion module effectively learns feature representations from global to local scales, ultimately enhancing small-target detection performance. Finally, downsampling operations are replaced with PSConv(Pinwheel-shaped Convolution), which better accommodates the Gaussian spatial pixel distributions of subtle small targets. Experimental results demonstrate that the proposed method achieves an mAP@0.5 of 97%, surpassing RT-DETR-r18 by 3%, and an mAP@0.5:0.95 of 53.4%, representing an improvement of 2.2%. Additionally, compared with the recently released YOLOv11m, our method improves mAP@0.5 by 5.6%. These results demonstrate the superior performance of the proposed method in PCB defect detection, which holds significant implications for industrial production. The code is available at https://github.com/Yttong-rr/SCPDETR/tree/master.

## 1. Introduction

Printed circuit boards (PCBs) are widely utilized in various electronic products. With rapid technological advancements, electronic devices are continuously trending towards miniaturization and lightweight designs, imposing increasingly stringent requirements on PCB manufacturing [1]. Serving as carriers for electronic components, PCBs containing defects such as short circuits and burrs [2] not only degrade the electronic performance of the boards but may also cause device malfunctions or even failure. Therefore, detecting these defects effectively is critically important. Nevertheless, The small size of defects on printed circuit boards (PCBs), combined with the densely arranged circuit layouts, results in a significant amount of interference information [3], making the identification of PCB defects challenging.

Traditional PCB defect detection primarily relies on manual inspection and electrical testing methods [4]. These two approaches suffer from low detection efficiency, poor detection accuracy, and the inability to perform real-time detection. Automated Optical Inspection (AOI) technology [5], due to its low cost and high efficiency, has become the mainstream approach for PCB quality inspection. Traditional AOI methods rely on machine learning techniques, such as Support Vector Machines (SVM) and Random Forests [6]. These methods perform well in feature extraction and classification but exhibit limitations when handling complex image data.

With the advancement of deep learning [7], the integration of AOI with deep learning algorithms has become the prevailing trend. Various object detection algorithms have emerged and been widely applied to PCB defect detection. Initially, two-stage networks, represented by R-CNN [8], were used. These networks first extract regions of interest in the first stage, followed by classification and bounding box regression in the second stage. Although effective, the two-stage computation makes them relatively slow and computationally expensive. Subsequently, one-stage algorithms, represented by the YOLO series [9], have been developed. These algorithms enable efficient and accurate detection of both surface and internal defects in PCBs [10].

The previous studies often rely excessively on complex network structures or specific attention mechanisms, and they still lack sufficient targeting and efficiency in the extraction of small-object features. Additionally, they overlook the capability of feature pyramids to capture small-object features, resulting in a detection accuracy that still has room for improvement [11].

After conducting an in-depth analysis of the limitations of these methods, we chose to focus on the critical step of feature extraction to enhance the accuracy of small-object recognition. In selecting a feature extraction method, we opted to improve the feature pyramid, as the backbone network, while primarily responsible for feature extraction and possessing strong feature learning capabilities, tends to lose detail information about small objects during the feature extraction process. In contrast, the feature pyramid method can effectively utilize feature maps at different scales, thereby better capturing the multi-scale features of small objects. Thus, this paper proposes an end-to-end detection network called SCP-DETR, based on improvements to the feature pyramid structure within the RT-DETR-R18 model. The primary contributions of this study can be summarized as follows:

1) We designed a unique fusion module, named CO-Fusion, which processes the received features at three different scales by dividing them into a global branch, a local branch, and a large branch. This approach generates richer and more accurate feature representations, thereby enhancing the ability to recognize and reconstruct objects and scenes in complex environments.
2) To further enhance the capability of small-object detection, we developed an SCP-DETR network based on RT-DETR-R18, which can effectively extract small-object information related to PCB defects.
3) We validate the proposed SCP-DETR network on the public dataset PKU-Market-PCB and compare its performance with other mainstream models. Experimental results demonstrate that our model achieves a 3% improvement in mAP compared to RT-DETR-R18 and surpasses the recently proposed YOLOv11n by 3.6% in terms of mAP.

## 2. Related work

Traditional PCB defect detection methods mainly include manual visual inspection and electrical testing. Manual visual inspection primarily involves workers examining PCBs using magnifying tools. However, modern PCBs are mass-produced in automated industrial workshops, making manual inspection unable to keep pace with production speed. Moreover, complex PCB backgrounds and extremely small defect sizes make manual defect detection challenging even with magnifying devices. This approach is time-consuming, labor-intensive, subjective, and prone to inaccuracies. Electrical testing typically involves using probes or contacts to measure electrical parameters on circuit boards [12]; defects are identified if measured parameters deviate from specified ranges. However, this method suffers from low efficiency, potential undetected defects, and possible damage to circuit boards.

Traditional methods struggle to meet the demands of mass production. Automatic Optical Inspection (AOI) using automated equipment has gradually become mainstream. Traditional AOI detection typically relies on machine learning algorithms. Zhang et al. [13] used template matching to locate defect regions, extracted geometric and histogram features, and classified defects using optimized Support Vector Machines (SVM). Lu et al. [14] extracted Histogram of Oriented Gradients (HOG) and Local Binary Patterns (LBP) features separately into SVM models, then fused these models based on Bayesian theory. Hagi et al. [15] accurately identified defect candidate regions through differences between test and reference images, then employed random sampling and feature selection for classification using SVM. Yuk et al. [16] extracted features using Speeded-Up Robust Features (SURF), trained fault patterns, and computed detection probabilities, forming Weighted Kernel Density Estimation (WKDE) maps to enhance inspection performance.

Many researchers have proposed deep learning techniques to improve small-object detection accuracy, enabling more effective capture of complex small-object features. Chen et al. [17] proposed LGCL-CenterNet, a lightweight network incorporating local-global context enhancement and dual-branch lightweight transformers. Jiang et al. [18] introduced RAR-SSD, incorporating multi-scale attention mechanisms and lightweight receptive field blocks (RFB-s) for broader effective feature extraction. Tang et al. [19] proposed PCB-YOLO based on YOLOv5, embedding Swin Transformer, K-means++, and EIoU loss to improve small-object localization. Ding et al. [20] introduced TDD-Net, leveraging multi-scale pyramid structures inherent in convolutional networks for PCB defect detection. Ji et al. [21] proposed MS-DETR, employing multi-stage convolution modules to enhance small-object feature extraction while reducing complexity. Du et al. [22] addressed the shortcomings of existing PCB surface defect detection methods, such as low accuracy and poor real-time performance, by proposing an enhanced YOLOv5s network named YOLO-MBBi for detecting surface defects on PCBs. Li et al. [23], aiming to accurately locate and recognize small objects, constructed a feature layer that integrates high-level semantic information with low-level geometric information. Wang et al. [24] proposed a lightweight printed circuit board surface defect recognition algorithm based on an improved YOLOv8-PCB. This algorithm introduces the C2f_SHSA attention mechanism into the backbone network, facilitating the lightweight and efficient fusion of local and global features.

In summary, the above methods all possess certain shortcomings. Manual and electrical inspections are inefficient and risk secondary damage. Machine-learning-based AOI requires manual feature engineering, making it labor-intensive and experience-dependent. Deep-learning-based AOI primarily focuses on feature extraction for small-object defects on PCBs. However, existing methods overly rely on complex network structures or specific attention mechanisms, neglecting the capability of feature pyramids to capture small objects effectively. Although detection accuracy has improved to some extent, there is still room for further enhancement. Therefore, this paper aims to achieve global modeling of complex images and precise localization of small-object defects by improving the feature pyramid.

## 3. Methodology

**Fig 1:**
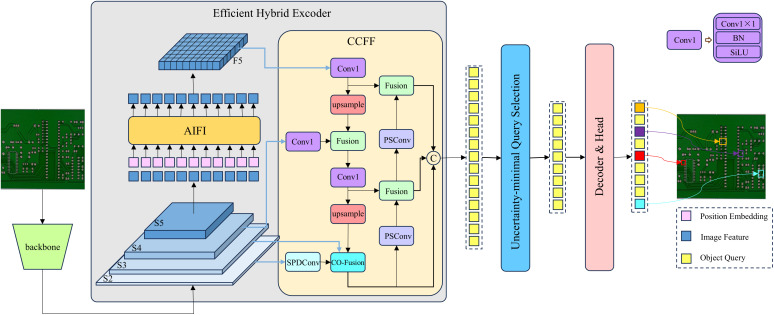
The overall architecture of SCP-DETR. (Compared with RT-DETR-R18, the SCP-DETR replaces the second Fusion module in the neck CCFF with our proposed CO-Fusion, incorporates the additional S2 feature layer processed by SPDConv, and replaces the downsampling operation with PSConv.).

Due to the dense circuit layouts of printed circuit boards (PCBs), the background tends to be highly complex, and defects are often extremely small and difficult to distinguish from background areas, resulting in significant interference and low detection accuracy [25]. To address these characteristics of PCB defects, we propose a novel network named SCP-DETR aimed at enhancing PCB defect detection accuracy. The overall architecture of the proposed model is illustrated in Fig 1. Our method is based on the RT-DETR-R18 architecture, with significant improvements made specifically to its CCFF neck module.Firstly, in order to fully capture small-object information, a common practice involves incorporating the shallow S2 feature layer into subsequent feature fusion processes. Shallow-layer features contain rich detail information and minimal distortion, which are crucial for preserving the edges of small defects. However, directly including the S2 feature layer introduces substantial computational overhead. To mitigate this issue, we introduce SPDConv(Space-to-depth Convolution) to efficiently process the S2 layer, effectively reducing computational complexity without information loss.Secondly, we fuse the processed S2 feature with S3 features and upper-layer features through our specifically designed CO-Fusion module. This module integrates these three feature scales, enabling comprehensive global modeling and thus providing richer and more robust feature representations for complex scene understanding.Finally, we replace the downsampling operation in the neck with PSConv(Pinwheel-shaped Convolution). By applying asymmetric padding, PSConv better aligns with the Gaussian spatial distribution characteristics of small targets [26], effectively aggregating central features over a wider receptive area and significantly enhancing the network’s ability to represent small defects.

### 3.1. Data preprocessing

To enhance the generalization performance and robustness of the model in complex scenarios, we employed a series of image preprocessing strategies during training. First, we used adaptive histogram equalization to dynamically improve image brightness, contrast, and clarity, making the targets in the images clearer and easier for the model to recognize. This method was fine-tuned to address uneven lighting issues commonly encountered during industrial image acquisition, effectively suppressing background noise. Notably, adaptive histogram equalization processes each region of the image independently, improving overall image quality while preserving local contrast. This enhances the prominence of detailed features, which is particularly critical for PCB defect detection. Secondly, we applied strategies such as horizontal flipping and random scaling to simulate various transformations that defect samples might undergo in real-world scenarios. This enabled the model to encounter a broader range of sample variations during training, thereby improving its adaptability to changes in perspective, size, and position. For the preprocessed images, we set the input size to 640 × 640, a choice that strikes a good balance between computational efficiency and accuracy.

### 3.2. SPDConv

For a typical backbone network, four stages of features are output, denoted as {S2, S3, S4, S5}. The shallow feature S2 contains fewer semantic details but is rich in fine-grained details and has minimal distortion. In contrast, the deep feature S5 is semantically richer but lacks fine details due to the progressive loss of information [27]. Most networks, including our chosen baseline network RT-DETR, typically incorporate the feature layers S3, S4, and S5 into the feature fusion process for further processing. However, small objects are more challenging to detect in the S3, S4, and S5 feature layers because detailed information is partially lost and resolution decreases after multiple stages of feature extraction.

Considering that PCB defects are predominantly small objects, we chose to incorporate the S2 feature layer into the neck-level feature fusion process to better capture fine-grained details for precise localization. To address the increased computational overhead caused by incorporating the S2 feature layer, we employed SPDConv [28] to reduce the computational complexity effectively. SPDConv internally maps spatial dimension information into the depth dimension, thus effectively reducing the size of the S2 feature map without losing spatial information. This approach prevents substantial increases in computational overhead and avoids excessive post-processing time associated with directly incorporating the S2 features.

**Fig 2:**
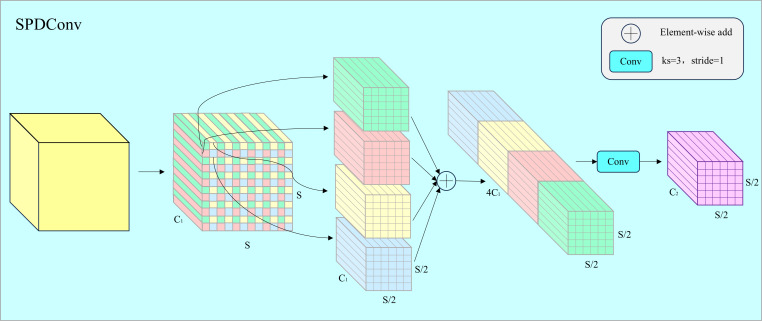
The detailed architecture of SPDConv with a scaling factor of 2.


The structure of SPDConv is shown in Fig 2. For an input feature X of arbitrary size $S\times S\times C_{1}$ , SPDConv first divides the feature into $scale^{2}$ sub-features based on a scaling factor denoted as scale. In this example, with scale = 2, the four resulting sub-features can be expressed as follows:

$$f_{0,0}=X\left[0:S:2,0:S:2\right],f_{1,0}=X\left[1:S:2,0:S:2\right]$$ (1)

$$f_{0,1}=X\left[0:S:2,1:S:2\right],f_{1,1}=X\left[1:S:2,1:S:2\right]$$ (2)

Here, **S** represents the width and height of the input feature map, so each sub-feature has a shape of $\left(\frac{S}{2},\frac{S}{2},C_{1}\right)$. The splitting operation is equivalent to a 2× down-sampling of the input feature map **X**. Next, we concatenate these four sub-features along the channel dimension, resulting in a feature map $X^{\prime }$. Consequently, the spatial dimensions of the resultant feature map $X^{\prime }$ are reduced to $\frac{1}{scale}$ of the original input feature map, while the channel dimension is increased to $scale^{2}$ times that of the original feature map. In other words, the original input feature map **X** with dimensions $\left(S,S,C_{1}\right)$ is transformed into an intermediate feature map $X^{\prime }$ with dimensions $\left(\frac{S}{scale},\frac{S}{scale},scale^{2}C_{1}\right)$.

After the feature transformation, a convolution with a kernel size of 3 ×3 and a stride of 1 is applied, reducing the final channel dimension to  $C_{2}<scale^{2}C_{1}$. Thus, the intermediate feature map $X^{\prime }(\frac{S}{scale},\frac{S}{scale},scale^{2}C_{1})$ is transformed into the final feature map $X^{\prime \prime }(\frac{S}{scale},\frac{S}{scale},C_{2})$. The reason for using a convolution with a stride of 1 is to preserve as much spatial discriminative feature information as possible. The SPD layer performs down-sampling on the input features while retaining all information in the channel dimension, ensuring no information loss. This allows the model to capture richer detail information at a reduced spatial resolution while preserving global contextual information.

### 3.3. CO-Fusion

**Fig 3:**
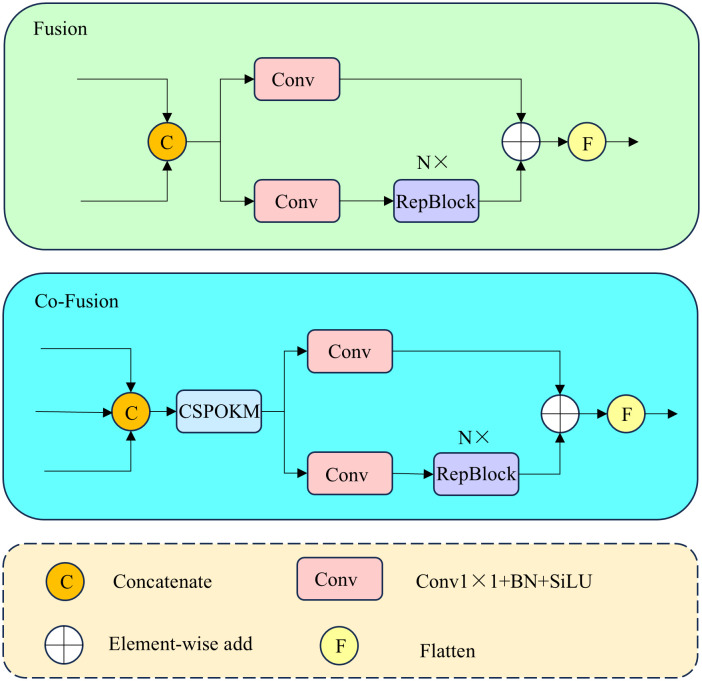
Structural comparison between the original Fusion module (top) and our proposed CO-Fusion module (bottom). In this paper, the number of RepBlocks is set to 3.

The original Fusion module employs only a single fusion operation, lacking the capability for global modeling during feature fusion. Consequently, this limitation negatively impacts the detection performance for small targets in complex PCB backgrounds. To address this, we replace the second Fusion module in the CCFF [29] neck with a newly designed CO-Fusion module, enabling better processing of the S2, S3, and upper-layer features. The structural comparison between the original Fusion module and our proposed CO-Fusion module is shown in Fig 3. The original Fusion module employs two 1 × 1 convolutions after concatenation to adjust the channel dimension, followed by N RepBlocks composed of RepConv units for deeper feature fusion. Outputs from two paths are combined via element-wise addition. However, this simple fusion approach struggles to capture global information from multiple feature scales. Therefore, we propose the CO-Fusion module, embedding our designed CSPOKM (CSP Omni-Kernel Module) after concatenation, allowing effective learning of feature representations from global to local scales.

**Fig 4:**
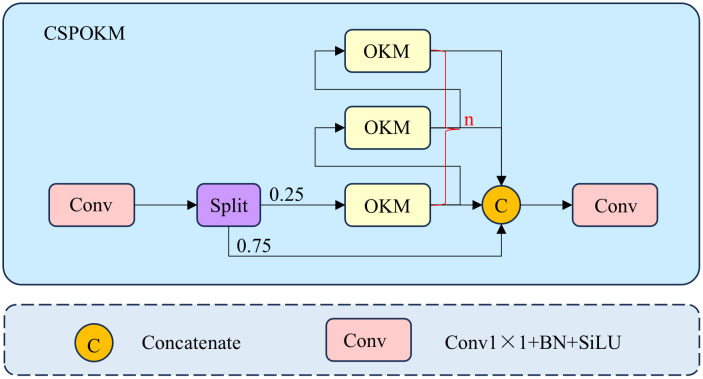
The structure diagram of CSPOKM. Here, *n* represents the number of OKMs used, and to reduce the number of parameters, ***n*** is set to 1 in this paper.

The CSPOKM integrates the Omni-Kernel Module (OKM) with the CSP structure, as illustrated in Fig 4. The input features are first processed by a 1 × 1 convolution and subsequently split along the channel dimension into two groups. One group, comprising 1/4 of the original channels, is fed into the OKM branch, which combines multi-level semantic information into richer and more accurate representations. The remaining 3/4 channels bypass any processing. Finally, these two feature groups are concatenated along the channel dimension and passed through another 1 × 1 convolution to adjust the output channels. To further reduce computational parameters, we set the number of OKM modules n = 1. By processing concatenated channel features, our CSPOKM module simultaneously perceives global, large-scale, and local features, enhancing the model’s multi-scale representational capabilities. Additionally, the CSP structure splits and partially reconnects features across stages, reducing redundancy in the channel dimension, enhancing feature representational power, and reducing convolutional parameters and computation.

**Fig 5:**
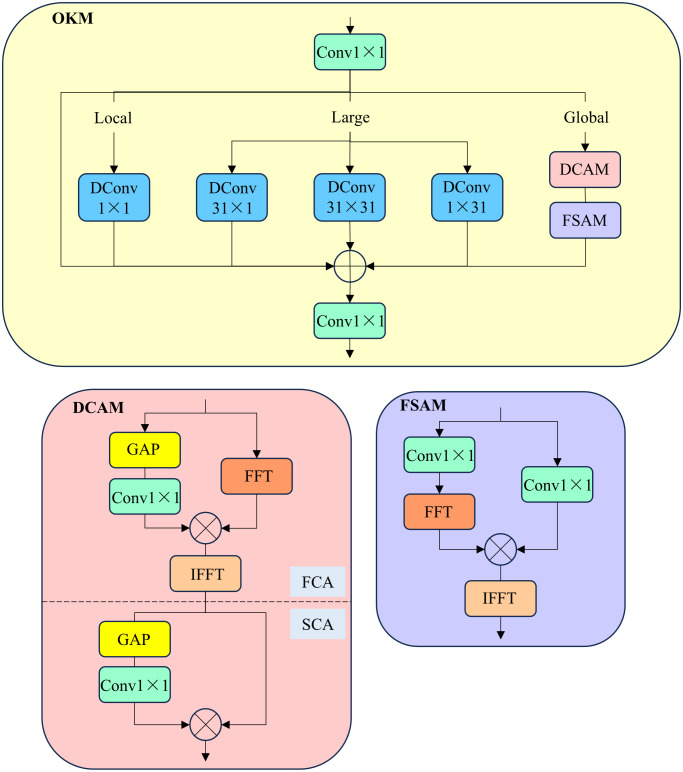
The overall structure of the Omni-Kernel Module. FFT and IFFT represent the Fast Fourier Transform and its inverse, respectively.

The structure of OKM [30] is shown in Fig 5. Given the input feature $X\in R^{C\times H\times W}$, it is first processed through a 1 × 1 convolution. The resulting features are then sent to three branches: the local branch, the large branch, and the global branch, to enhance the model’s multi-scale representation and improve its ability to model the original complex image. The outputs from the three branches are element-wise summed and then passed through another 1 × 1 convolution to adjust the number of channels.

#### 3.3.1. Large branch.

In the large branch, a cheap depthwise convolution with a kernel size of K × K is applied to obtain a large receptive field. Additionally, 1 × K and K × 1 depthwise convolutions are used to capture strip-like contextual information. To avoid the massive computational overhead introduced by large-kernel convolutions, this paper replaces only the second Fusion module with CO-Fusion, and only one OKM is employed in the OKM branch of CSPOKM. Finally, based on information about receptive field size, image resolution, and computational complexity, this paper selects K = 31 for the kernel size in the large branch. The outputs of the K × K, 1 × K, and K × 1 convolutions in the large branch are then summed to capture large-scale spatial information.

#### 3.3.2. Global branch.

However, the large branch is trained on preprocessed cropped images during training, while during inference, the input images are larger than the cropped images used during training. Therefore, a 31 × 31 convolution kernel cannot cover the global features. To address this issue, the global branch applies dual-domain processing to enhance global modeling capability. The global branch consists of the Dual-domain Channel Attention Module (DCAM) and the Frequency-based Spatial Attention Module (FSAM). In DCAM, for the input feature $X_{Global}\in R^{C\times H\times W}$, the feature is first processed through the Frequency Channel Attention (FCA) module, and the FCA process is described by the following formula:


$$X_{FCA}=IF(F(X_{Global})⊗W_{1\times 1}^{FCA}(GAP(X_{Global}))$$ (3)

Here, F and IF represent the Fast Fourier Transform (FFT) and its inverse operation, respectively. $X_{FCA}$ denotes the output features processed by the FCA module, $X_{Global}$ refers to the features input to the global branch, ⊗ represents element-wise multiplication, $W_{1\times 1}^{FCA}$ refers to the 1 × 1 convolution layer in the FCA module, and GAP stands for global average pooling. By leveraging the Fourier Transform technique and effectively refining global characteristics in the frequency domain based on the Convolution Theorem, the global features are enhanced. The resulting features are then further sent to the Spatial-Channel Attention (SCA) module within DCAM. Therefore, the overall process of DCAM can be expressed as:


$$X_{DCAM}=X_{FCA}⊗W_{1\times 1}^{SCA}(GAP(X_{FCA}))$$ (4)

Here, $X_{DCAM}$ represents the output features processed by DCAM, $X_{FCA}$ denotes the output features from the FCA module, ⊗ represents element-wise multiplication, $W_{1\times 1}^{SCA}$ refers to the 1 × 1 convolution in the SCA module, and GAP stands for global average pooling. DCAM enhances dual-domain features in a coarse-grained manner along the channel dimension. Subsequently, we apply the Frequency-based Spatial Attention Module (FSAM) in the spatial dimension to refine the spectrum at a fine-grained level. The FSAM process is expressed by the following formula:


$$X_{FSAM}=IF(F(W_{1\times 1}^{1}(X_{DCAM}))⊗W_{1\times 1}^{2}(X_{DCAM}))$$ (5)

Here, $X_{FSAM}$ represents the output features of the FSAM module, F and IF represent the Fast Fourier Transform (FFT) and its inverse operation, respectively. $W_{1\times 1}^{1}$ and $W_{1\times 1}^{2}$ refer to the 1 × 1 convolution operations on both branches, and $X_{DCAM}$ is the output feature of the DCAM module. ⊗ denotes element-wise multiplication. Through DCAM and FSAM, this global branch focuses on the frequency information relevant to high-quality image reconstruction.

#### 3.3.3. Local branch.

In addition to the large branch and global branch, which capture larger receptive fields, local information plays a crucial role in image restoration. Therefore, a very simple yet effective local branch is designed, which uses a 1 × 1 depthwise convolutional layer for local signal modulation to capture fine-grained local details, complementing small-scale features.

Finally, OKM, consisting of the global branch, large branch, and local branch, forms a full-kernel module capable of effectively learning feature representations from global to local scales.

### 3.4. PSConv

Currently, mainstream downsampling methods include pooling [31] and convolutional operations [32]. Common pooling methods, such as max-pooling and average-pooling, although simple and low in computational cost, may lose important boundary and texture information or blur features, damaging structural integrity. Convolutional downsampling typically employs stride-2 convolutions, which do not consider the spatial pixel distribution of small targets and incur higher computational overhead. The original downsampling used in RT-DETR’s CCFF employs conventional 3 × 3 convolutions with stride 2. Thus, taking into account the characteristics of small PCB defect targets and complex backgrounds, we replace the conventional convolution in downsampling with PSConv [33]. PSConv aligns with the Gaussian spatial distribution of small targets, significantly enhancing feature extraction, enlarging receptive fields, and reducing computational parameters.

**Fig 6:**
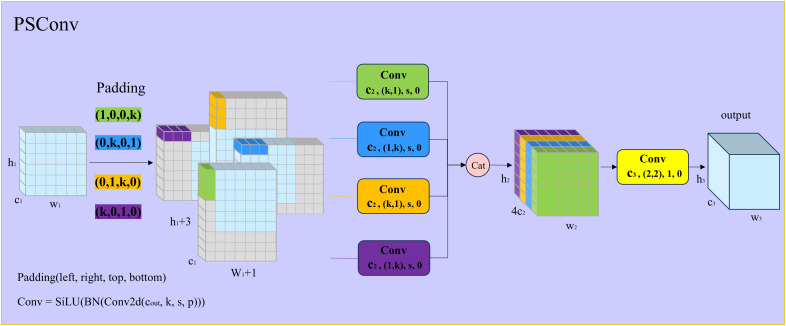
The overall architecture of the pinwheel-shaped convolutional module. Here, *k* represents the size of both the surrounding padding and the convolutional kernel, which is set to 3 in this paper. *s* denotes the stride size, with a value of 2 in this work.


In traditional convolutional layers, fixed-shaped convolution kernels are typically used, which are often ineffective in capturing the fine-grained features of small objects. In contrast, PSConv leverages asymmetric padding and windmill-shaped convolution kernels to better align with the imaging characteristics of targets, thereby enhancing the ability to extract low-level features. The structure of PSConv is illustrated in Fig 6. For an input feature map with a shape of $X^{\left(h_{1},w_{1},c_{1}\right)}$, where $h_{1},w_{1},c_{1}$ represent the height, width, and number of channels of the feature map, respectively, PSConv achieves feature fusion in four different directions by applying different padding to the feature space (left, right, top, and bottom) and creating horizontal and vertical convolution kernels for these padded regions. The operation of the first layer of parallel convolutions in PSConv can be expressed as:

$$X_{1}^{\left(h_{2},w_{2},c_{2}\right)}=SiLU(BN(X_{P(1,0,0,k)}^{\left(h_{1},w_{1},c_{1}\right)}⊗W_{1}^{\left(k,1,c_{2},s,0\right)}))$$ (6)

$$X_{2}^{\left(h_{2},w_{2},c_{2}\right)}=SiLU(BN(X_{P(0,k,0,1)}^{\left(h_{1},w_{1},c_{1}\right)}⊗W_{2}^{\left(1,k,c_{2},s,0\right)}))$$ (7)

$$X_{3}^{\left(h_{2},w_{2},c_{2}\right)}=SiLU(BN(X_{P(0,1,k,0)}^{\left(h_{1},w_{1},c_{1}\right)}⊗W_{3}^{\left(k,1,c_{2},s,0\right)}))$$ (8)

$$X_{4}^{\left(h_{2},w_{2},c_{2}\right)}=SiLU(BN(X_{P(k,0,1,0)}^{\left(h_{1},w_{1},c_{1}\right)}⊗W_{4}^{\left(1,k,c_{2},s,0\right)}))$$ (9)

where ⊗ represents the convolution operation. To improve training stability and speed, batch normalization (BN) and the Sigmoid activation function are applied after each convolution. $W_{1}^{\left(k,1,c_{2},s,0\right)}$ is a convolution kernel with an output channel size of $c_{2}$, a kernel size of k × 1, a stride of s, and no padding. In this study, k is set to 3, and s is set to 2. The padding parameter P(1,0,0,k) specifies the number of pixels padded in the left, right, top, and bottom directions, respectively. The relationship between the width, height, and number of channels of the output features after the first layer of convolution and those of the input features is given by:


$$h_{2}=\frac{h_{1}}{s}+1,w_{2}=\frac{w_{1}}{s}+1,c_{2}=\frac{c_{3}}{4}$$ (10)

Where $c_{3}$ is the total number of output channels after applying PSConv. The features obtained from the above operation are then concatenated along the channel dimension, which can be expressed as:


$$\begin{array}{c} X^{\prime (h_{2},w_{2},4c_{2})}=Cat(X_{1}^{\left(h_{2},w_{2},c_{2}\right)},...,X_{4}^{\left(h_{2},w_{2},c_{2}\right)}) \end{array}$$ (11)

Finally, a convolution operation is applied to normalize the concatenated features, and the final output features Y can be expressed as:


$$Y^{\left(h_{3},w_{3},c_{3}\right)}=SiLU(BN(X^{\prime (h_{2},w_{2},4c_{2})}⊗W^{\left(2,2,c_{3},1,0\right)}))$$ (12)

$$h_{3}=h_{2}-1=\frac{h_{1}}{s},w_{3}=w_{2}-1=\frac{w_{1}}{s}$$ (13)

where $W^{\left(2,2,c_{3},1,0\right)}$ represents a convolution kernel with a size of 2 × 2, an output channel size of $c_{3}$, a stride of 1, and no padding. The width and height of the final output features are consistent with those of the input features, which allows PSConv to be seamlessly substituted for standard convolutional layers.

**Fig 7:**
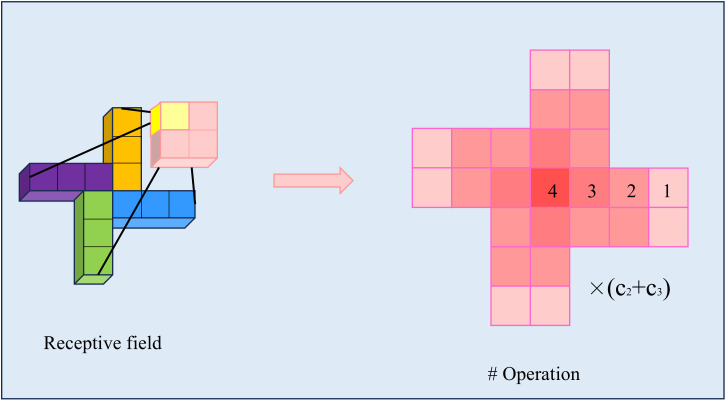
The effective receptive field of PSConv when *k* is 3. The shades of red represent the effectiveness of the receptive field.


PSConv applies asymmetric padding to features, and its final receptive field is shown in Fig 7. This illustrates that when k = 3, the range of its receptive field is 25. The depth of the red color represents the effectiveness of the receptive field, where it can be observed that the effectiveness gradually decreases outward, while the center point exhibits the highest effectiveness. This distribution is similar to a Gaussian distribution, and the operation of concentrating features at the center aligns with the characteristics of small object distributions.In addition, PSConv operates by dividing the convolution into four grouped convolutions, which not only reduces the computational cost of parameters but also achieves a larger receptive field. The parameter calculation formulas for standard convolution and PSConv are as follows:

$$Conv_{para}=c_{3}\times c_{1}\times k,(bias=False)$$ (14)

$$PSConv_{para}=4\times (\frac{c_{3}}{4}\times c_{1}\times k\times 1)+c_{3}\times c_{1}\times 4=(k+4)\times c_{3}\times c_{1}$$ (15)

In this study, *k* represents the size of the convolutional kernel, which is set to 3. Additionally, PSConv is used to replace the two down-sampling operations in the neck, ensuring that the number of output feature channels is equal to the number of input feature channels. As a result, the parameter count of a standard convolution is $9c_{1}^{2}$, while the parameter count of PSConv is $7c_{1}^{2}$. Therefore, when the convolutional kernel size is 3, the parameter count is reduced by 22.2%, while the receptive field is increased by 177%.

## 4. Experiment

### 4.1. Dataset

**Fig 8:**
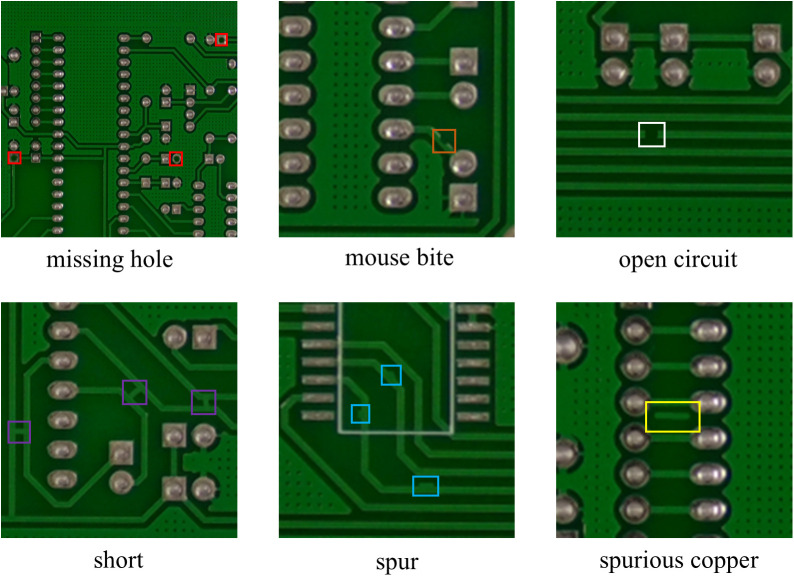
The six PCB defect categories in the PKU-Market-PCB dataset. Since the target defects are too small, the images have been magnified and cropped for better visualization of the defects.

The dataset utilized in this study is the Printed Circuit Board (PCB) Defect Dataset [34] (PKU-Market-PCB), which was released by the Intelligent Robotics Open Laboratory of Peking University. This dataset is a publicly available synthetic PCB dataset containing 1,386 images. The original dataset is designed for detection, classification, and registration tasks. For this study, we selected 693 images specifically intended for detection tasks. The original dataset labels are in XML format, and the image resolutions primarily fall into four specifications: 3034 × 1586, 2904 × 1921, 3056 × 2464, and 2282 × 2248. The images have a DPI of 72. A key characteristic of this dataset is that the number of images is relatively evenly distributed across different defect categories, and the defects pertain to small-object defect detection. This dataset includes six different types of PCB defects: missing hole, mouse bite, open circuit, short, spur, and spurious copper. Fig 8 illustrates typical sample images of these six types of PCB defects. To better showcase the sample images, the original images were enlarged and cropped for enhanced visualization. From the figure, it is evident that the sizes of the defects are very small; even after enlargement and cropping, it is difficult to identify the defect locations without close inspection. Moreover, the background texture of PCBs is highly complex [35], consisting primarily of various traces, pads, and vias. This results in a low degree of distinguishability between the target defects and the background, as the extracted features contain a significant amount of interference information. This makes it challenging to differentiate target defect information from background noise, posing substantial difficulties for model recognition.

**Fig 9:**
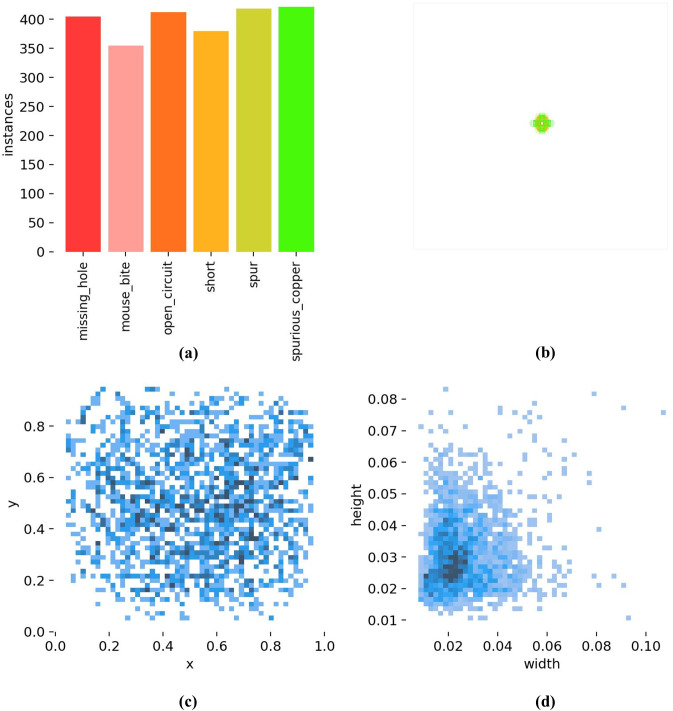
The label distribution of the PKU-Market-PCB dataset; (a) Statistical chart of the six defect categories. (b) Distribution of bounding box sizes in the dataset. (c) Distribution of object center points relative to the entire image. (d) Aspect ratio distribution of target objects relative to the whole image.

Fig 9 shows the detailed label distribution of the six defect categories in the PKU-Market-PCB dataset. From Fig 9(a), it can be observed that the dataset maintains a roughly balanced distribution across the six defect categories, with each category containing approximately 400 samples. Such a balanced distribution is advantageous for training the model to learn comprehensive and well-rounded information. Fig 9(b) depicts the size distribution of the bounding boxes for the target defects. Relative to the entire image, the defect sizes occupy only a small portion, indicating that most defects in PCB images fall into the category of small objects. In deep learning-based object detection tasks, small objects generally refer to targets with relatively small sizes and low pixel proportions within the image. Due to the unique characteristics of PCB manufacturing processes, the defect sizes are inherently small, which places higher demands on the model’s ability to detect small objects. The spatial distribution of the center points of all target objects within the dataset is shown in Fig 9(c). Most defects are concentrated near the center of the image, which corresponds to the layout design of PCBs, where the circuit traces are typically distributed in the central region. Fig 9(d) illustrates the aspect ratio of target objects relative to the overall image. Most targets are concentrated in the lower left corner, where the width and height ratios relative to the image are approximately 0.03. This further demonstrates that most PCB defects are small objects, emphasizing the need for the model to focus on detecting small targets effectively.

To improve the model’s performance and optimize the dataset partitioning strategy, it is essential to gain an in-depth understanding of the dataset’s distribution characteristics. Based on this, we partitioned the selected dataset into training and validation sets at a ratio of 9:1. The final training set contains 554 images, and the validation set contains 139 images. Additionally, we selected 130 images from the remaining data in the original dataset as a test set to evaluate the model’s detection performance. We further reviewed the selected samples to ensure that their distribution is uniform and representative of the overall dataset. This strategy mitigates potential biases caused by improper dataset partitioning, thereby enhancing the robustness of the experimental results.

### 4.2. Evaluation metrics

To objectively evaluate the performance and effectiveness of the model, we selected Precision, Recall, mAP@0.5, mAP@0.5:0.95, Parameters, and Gflops [36] as evaluation metrics to comprehensively assess the model’s capabilities. Precision, Recall, mAP@0.5, and mAP@0.5:0.95 are used to reflect the accuracy of the model in target recognition. Among them, Precision indicates the proportion of correctly identified targets among all the targets predicted by the model. Recall indicates the proportion of correctly identified targets among all true targets [37]. mAP@0.5 represents the mean Average Precision (AP) calculated for each PCB defect category with an IoU threshold set to 0.5. mAP@0.5:0.95 represents the mean AP calculated across IoU thresholds ranging from 0.5 to 0.95. Parameters and Gflops are used to evaluate the model’s real-time performance and complexity, Parameters measure the size and complexity of the model. Gflops measure the computational cost and execution time of the model. The formulas for calculating these metrics are as follows:


$$Precision=\frac{TP}{TP+FP}$$ (16)

$$Recall=\frac{TP}{TP+FN}$$ (17)

$$AP=\int_{0}^{1}P\left(r\right)dr$$ (18)

$$mAP=\frac{\sum_{i=1}^{N}AP_{i}}{N}$$ (19)

Among them, TP refers to true positives, which are positive samples correctly predicted as positive by the model. FP refers to false positives, which are negative samples incorrectly predicted as positive by the model. FN refers to false negatives, which are positive samples incorrectly predicted as negative by the model [23]. *P(r)* represents the smoothed precision-recall curve, and AP (Average Precision) is the area under the *P(r)* curve obtained through integration. *N* represents the number of defect categories, which in this study corresponds to the six PCB defect categories.

### 4.3. Implementation details

In this work, our improved algorithm is based on the RT-DETR-R18 model and was further refined for experiments conducted on the printed circuit board (PCB) defect dataset. The hyperparameters used for the model in this experiment are shown in Table 1.

**Table 1:** Training parameters.

Parameter	Value
Image Size	640 × 640
Learning Rate	0.0001 ~ 1
Weight Decay	0.0001
Momentum	0.9
Optimizer	AdamW
Mosaic	0
Mixup	0
Batch Size	8
Epoch	300

It is important to note that we did not use Mosaic or MixUp data augmentation during training. Although enabling these augmentation techniques might improve accuracy, the augmented images differ from real-world data, and we focused solely on improving the model itself to enhance accuracy. All experiments in this study were conducted on an Ubuntu 20.04 operating system. The selected environment included Python 3.8, PyTorch 2.0.0, and CUDA 11.8. The hardware used consisted of an NVIDIA RTX 3090 GPU with 24 GB of VRAM, a 10-core CPU, and 30 GB of system memory.

### 4.4. Ablation experiment

To validate the effectiveness of each enhancement component in SCP-DETR, we conducted independent ablation experiments on the dataset. The experimental results are shown in Table 2. The components are as follows: SPDConv, a spatial-to-depth convolution designed to reduce the parameters introduced by the addition of the S2 feature layer while retaining fine-grained information; CO-Fusion, a fusion module designed to effectively learn feature representations from global to local through different branches; and PSConv, a pinwheel-shaped convolution that replaces the original down-sampling operation. The baseline network used is RT-DETR-r18.

**Table 2:** Ablation study of each component in SCP-DETR.

RT-DETR	SPDConv	CO-Fusion	PSConv	P(%)	R(%)	mAP @0.5(%)	mAP @0.5:0.95(%)	Para	Gflops
√	–	–	–	0.851	0.98	0.94	0.512	20089448	58.3
–	√	–	–	0.855	0.99	0.959	0.516	20252776	61
–	–	√	–	0.837	0.99	0.969	0.525	20444008	63.2
–	–	–	√	0.858	0.98	0.955	0.533	19631208	57.8
√	√	√	√	0.865	0.99	0.97	0.534	20247656	66.1

1) **Effectiveness of SPDConv**. SPDConv completely eliminates the strided convolution and pooling operations in traditional convolution, reducing the loss of fine-grained features. By employing spatial-to-depth convolution, SPDConv reduces the spatial resolution of feature maps without losing spatial information, preserving discriminative features while decreasing the computational cost of subsequent convolution operations. By processing the S2 feature layer with SPDConv and incorporating it into subsequent fusion operations, the computational cost of directly adding S2 to subsequent operations using traditional approaches is significantly reduced. SPDConv enables the capture of richer detail information at a smaller spatial resolution while retaining global contextual information. Compared with RT-DETR, incorporating SPDConv increases Precision by 0.4%, Recall by 1%, and mAP@0.5 by 1.9%, with a negligible increase in parameter count, demonstrating the effectiveness of SPDConv.
2) **Effectiveness of CO-Fusion**. CO-Fusion incorporates the CSPOKM module, designed by us, after the concatenation operation in the original fusion module. This allows the model to simultaneously perceive global, mid-scale, and local information, achieving richer feature representations and enhancing the ability to model complex scenarios. Its local branch captures fine-grained details through 1 × 1 convolution, improving the model’s ability to detect small target edge details. Furthermore, by combining it with the CSP structure, redundant information in the channel dimension is reduced, thereby enhancing feature representation. Incorporating CO-Fusion increases Recall by 1%, mAP@0.5 by 2.9%, and mAP@0.5:0.95 by 1.3%, with negligible increases in parameter count and computational cost. These results strongly demonstrate the critical role of CO-Fusion in the model.
3) **Effectiveness of PSConv**. PSConv introduces different paddings for the input features and stitches them together through convolution operations, resulting in an expanded windmill-shaped receptive field. This shape aligns with the Gaussian spatial distribution of small target pixels. By replacing the down-sampling operation with PSConv, the model can aggregate information over a larger area while maintaining a lightweight design, improving the ability to model PCB circuit diagrams and better capture defect information. Compared with RT-DETR, incorporating PSConv increases Precision by 0.7%, mAP@0.5 by 1.5%, and mAP@0.5:0.95 by 2.1%, while reducing the parameter count by 458,240, demonstrating the effectiveness and lightweight nature of PSConv.

By integrating SPDConv, CO-Fusion, and PSConv into RT-DETR-r18, we ultimately construct our improved network, SCP-DETR. This results in increases in Precision and Recall by 1.4% and 1%, respectively, mAP@0.5 by 3%, and mAP@0.5:0.95 by 2.2%. The increase in parameter count and computational cost is minimal, achieving better results with nearly the same computational budget. These findings further confirm the effectiveness of the three components for PCB defect detection.

### 4.5. Comparative test

In this section, we conduct comparative experiments to demonstrate the superiority of our improved SCP-DETR network. We compare it with several state-of-the-art object detection algorithms on the PCB defect dataset, including multiple versions of YOLO (YOLOv8, YOLOv9, YOLOv10, YOLOv11) and RT-DETR. The experimental results are shown in Table 3. SCP-DETR achieves higher mAP@0.5 than all other networks, with an improvement of 1.1%−6.9%. For mAP@0.5:0.95, SCP-DETR falls short of YOLOv9m by only 1% but outperforms the remaining methods by 0.2%−5.2%. Recall improves by 0%−7%. Notably, compared to RT-DETR-r50, the proposed model achieves a 1.3% improvement in Precision and a 1.6% improvement in mAP@0.5, while its parameter count and computational cost are only half of RT-DETR-r50. Furthermore, compared to the latest YOLOv11m, SCP-DETR improves Precision and Recall by 4.2% and 7%, respectively, mAP@0.5 by 5.6%, and mAP@0.5:0.95 by 3.2%, with similar computational overhead. By comparing the results with those of previous studies, we found that SCP-DETR achieves a higher mAP@0.5 than all other approaches. Furthermore, we observed that the Recall of SCP-DETR is also higher than that of other methods. Previous methods often rely excessively on complex network structures or specific attention mechanisms, and their effectiveness and efficiency in extracting small object features remain inadequate. In contrast, SCP-DETR improves upon the feature pyramid, enabling the effective utilization of feature maps at different scales, thereby better capturing the multi-scale features of small objects. Consequently, the experimental results demonstrate the model’s capability in detecting real-world defects, as it can comprehensively identify all targets with greater accuracy.

**Table 3:** Performance comparison between SCP-DETR and various mainstream object detection algorithms.

Method	P(%)	R(%)	mAP @0.5(%)	mAP @0.5:0.95(%)	Parameters	Gflops
YOLOv8s	0.897	0.94	0.928	0.492	11137906	28.7G
YOLOv8m	0.932	0.95	0.939	0.51	25843234	78.7
YOLOv8l	0.937	0.96	0.955	0.52	43611234	164.8
YOLOv9s	0.839	0.96	0.959	0.522	9747236	39.6
YOLOv9m	0.862	0.96	0.956	0.544	32771428	132.4
YOLOv10n	0.899	0.94	0.905	0.482	2709380	8.4
YOLOv10s	0.891	0.93	0.901	0.484	8070996	24.8
YOLOv10m	0.898	0.95	0.929	0.501	16491076	64
YOLOv11n	0.802	0.94	0.934	0.514	2591010	6.4
YOLOv11s	0.793	0.92	0.919	0.513	9430114	21.6
YOLOv11m	0.823	0.92	0.914	0.502	20057634	68.2
RT-DETR-r18	0.851	0.98	0.94	0.512	20089448	58.3
RT-DETR-r50	0.852	0.99	0.954	0.532	42792550	134.5
RT-DETR-l	0.873	0.99	0.958	0.532	32818662	108
YOLO-MBBi [22]	0.958	0.946	0.953	–	–	12.8
Li et al. [23]	–	–	0.962	–	13700000	–
YOLOv8-PCB [24]	0.947	0.94	0.961	–	2460000	7.1
**SCP-DETR**	**0.865**	**0.99**	**0.97**	**0.534**	**20247656**	**66.1**

In summary, the SCP-DETR model demonstrates strong competitiveness and significant potential for application in PCB defect detection in terms of detection accuracy and computational complexity.

**Fig 10:**
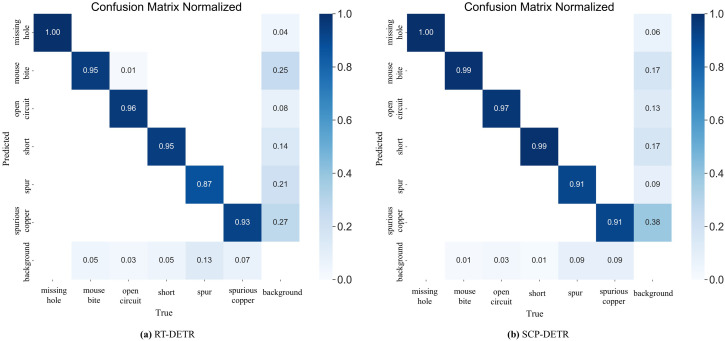
Comparison of normalized confusion matrices between RT-DETR and SCP-DETR on the PKU-Market-PCB dataset.

The confusion matrix is a critical tool for evaluating model performance. By comparing the model’s predictions with the ground-truth labels, various evaluation metrics, such as accuracy, recall, AP, and mAP, can be calculated. Fig 10 presents the normalized confusion matrices of RT-DETR and SCP-DETR on the PKU-Market-PCB dataset during testing. The horizontal axis of the matrix represents the ground-truth classes, while the vertical axis represents the predicted classes. Elements along the main diagonal of the matrix correspond to the number of correctly detected samples. The lower left triangular region indicates the number of missed detections, also known as false negatives, while the upper right triangular region represents the number of false detections, also known as false positives.

From the figure, it can be observed that, except for the spurious copper category, SCP-DETR achieves higher prediction accuracy across all other categories compared to RT-DETR. This indicates that SCP-DETR exhibits some limitations in feature extraction for the spurious copper category. Notably, the accuracy of both models for the spurious copper category is relatively low. This is primarily due to the fact that this category has minimal distinction from background elements, making it challenging for the models to differentiate. As a result, the detection performance for this category is somewhat inadequate.

**Fig 11:**
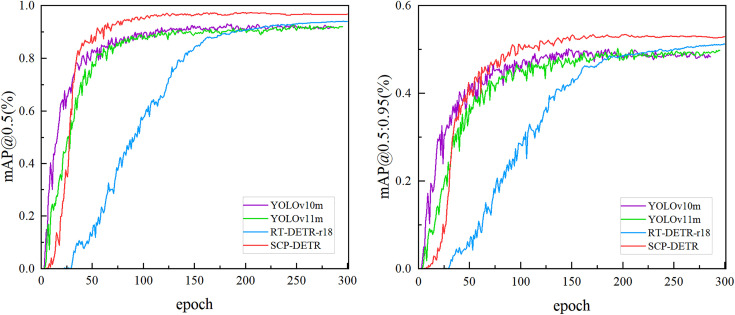
Comparison of SCP-DETR and other models on the PKU-Market-PCB dataset during training in terms of mAP@0.5 (left) and mAP@0.5:0.95 (right).

To observe the changes in accuracy during the training process, we visualized the mAP@0.5 and mAP@0.5:0.95 curves over training epochs for YOLOv10m, YOLOv11m, RT-DETR-r18, and SCP-DETR, as shown in Fig 11. From the figure, it can be seen that YOLOv10m achieves higher mAP@0.5 and mAP@0.5:0.95 than the other networks within the first 25 epochs, while SCP-DETR lags behind YOLOv10m and YOLOv11m during this period. However, after 50 epochs, SCP-DETR surpasses all other models and ranks first. By the end of 300 training epochs, all networks stabilize, with SCP-DETR significantly outperforming the others, RT-DETR-r18 ranking second, and the curves of YOLOv10m and YOLOv11m nearly overlapping with minor differences.

**Fig 12:**
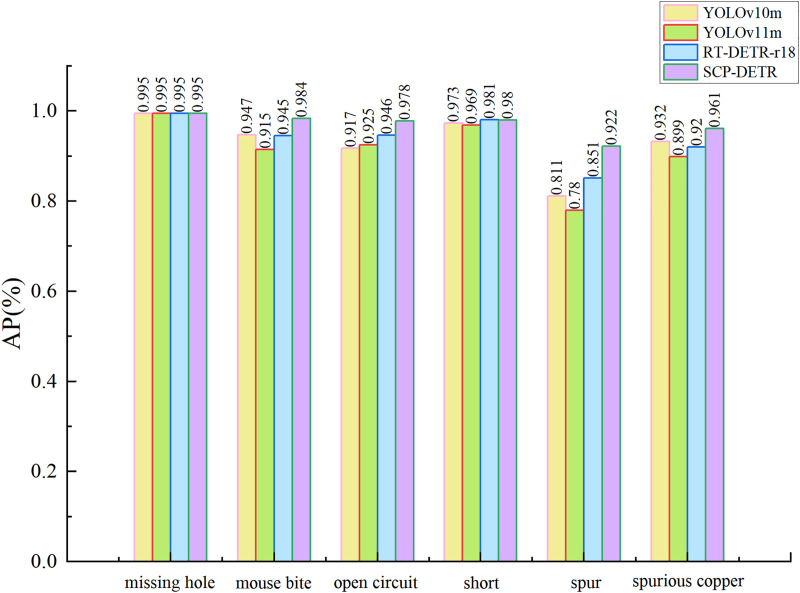
The prediction accuracy bar chart of SCP-DETR and other models on the six PCB defect categories in the PKU-Market-PCB dataset.

Fig 12 presents a comparison of AP for each PCB defect category between SCP-DETR, YOLOv10m, YOLOv11m, and RT-DETR-r18. From the figure, it is evident that, except for the “short” category where SCP-DETR’s AP is 0.1% lower than RT-DETR, SCP-DETR achieves AP values that are either higher than or on par with the other three networks across all other categories. It is also clear from the figure that “missing hole” is the easiest category to predict, with all networks maintaining an AP of 99.5%. This is because, in the PCB background, which predominantly consists of green circuit board layouts, the “missing hole” defect is located on the pads, creating a sharp contrast in color with the green background, making it the easiest to recognize. Conversely, “spur” is the most challenging category to detect, with the largest AP variation across networks. This is likely due to its smaller size compared to other defect types and its low distinguishability from the background, making it difficult to focus on in complex backgrounds. For this category, SCP-DETR achieves an AP that is 21.2% higher than the lowest-performing YOLOv11m and 14.1% higher than RT-DETR-r18. These results highlight SCP-DETR’s superior ability to detect small objects in complex scenarios.

### 4.6. Qualitative results

**Fig 13:**
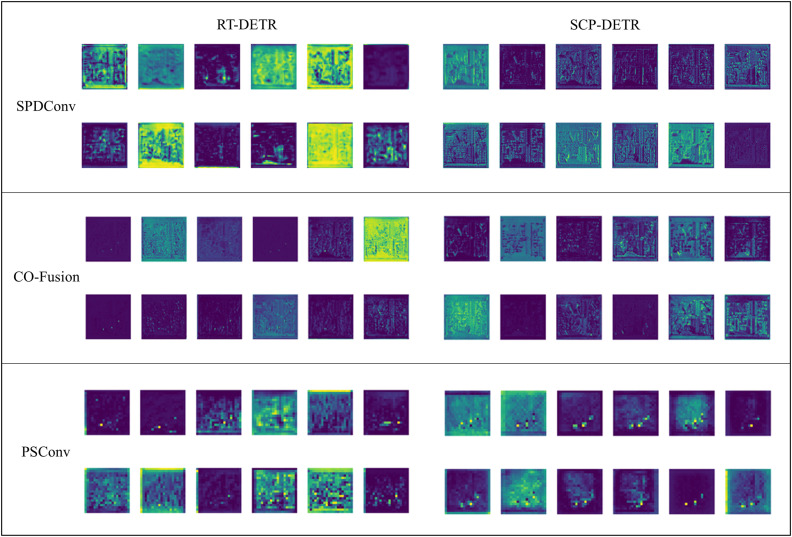
Visualization comparison of feature maps for 12 channels after adding modules during the feature extraction stage in RT-DETR and SCP-DETR.

To intuitively demonstrate the effects of each enhancement component in SCP-DETR, we visualized and compared the feature maps of RT-DETR with those of SCP-DETR after integrating the three enhancement modules. Each part displays 12 channels of feature maps, as shown in Fig 13. For SPDConv, which is applied to process the S2 feature layer, we selected the processed S2 features, S3 features, and the concatenated features of the previous layer for visualization. From the figure, it is evident that after incorporating SPDConv, the feature maps of the improved model are more three-dimensional and clearer, whereas RT-DETR has already blurred the edge detail information, which is highly detrimental for the localization of small targets. CO-Fusion is used to fuse S2 features, S3 features, and the features of the previous layer. Therefore, we selected the final feature maps processed by CO-Fusion and the second Fusion module for comparison. The feature maps of RT-DETR, which use the original Fusion module, are highly flattened, with almost no visible information about the circuit layout. However, after replacing the Fusion module with CO-Fusion, the resolution of the feature maps significantly improves, and the features become more three-dimensional and detailed, allowing the original circuit layout to be roughly discerned. This demonstrates that CO-Fusion reduces feature loss and exhibits a strong ability to model complex backgrounds. Finally, PSConv replaces two down-sampling operations in the neck of the network. The original RT-DETR uses a convolution with a kernel size of 3 and a stride of 2. We selected the feature maps from the final replacement location for comparison. At this stage, the feature maps have undergone many operations, which have already caused the loss of substantial detail information. RT-DETR, using standard convolution, can no longer distinguish background information from defect information. In contrast, after replacing the operation with PSConv, it is still possible to clearly distinguish three defect regions and accurately locate them while separating them from the background information. Through the visualization of these feature maps, the superiority of our proposed enhancements in feature processing is demonstrated.

**Fig 14:**
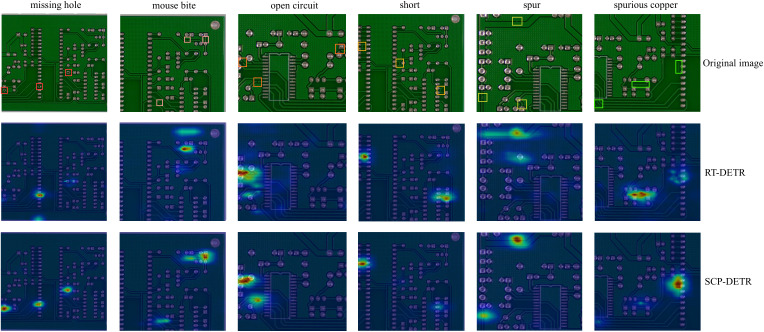
Heatmap comparison of detection results for the six PCB defect categories between RT-DETR and SCP-DETR networks. The dark red areas indicate regions that the models focus on.

Fig 14 compares the heatmaps of RT-DETR and SCP-DETR across six defect categories to analyze the differences in attention between our proposed model and the original model. In these heatmaps, deep red regions indicate areas the model focuses on, which play a crucial role in defect localization and classification. For the missing hole, mouse bite, short, and spurious copper categories, while RT-DETR does focus on some defects, it also significantly focuses on unnecessary background information above or below the defects. In some cases, it entirely neglects the defect areas. In contrast, SCP-DETR’s attention is concentrated on the defect regions. For the open circuit category, RT-DETR fails to focus on the defect in the upper-right corner, and its localization for the defect in the lower-left corner is inaccurate and scattered. For the spur category, neither model focuses on the defect at the very bottom, possibly due to the defect being too small. Furthermore, RT-DETR primarily focuses on the pad information above, which could lead to false positives. In conclusion, SCP-DETR can accurately localize defect positions and distinguish them from background information.

**Fig 15:**
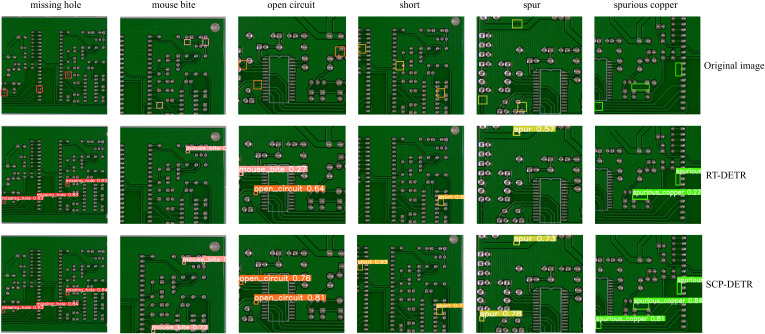
Detection results of RT-DETR and SCP-DETR on six PCB defect categories.

To further illustrate the detection performance of our improved model, Fig 15 shows the detection results of RT-DETR and SCP-DETR on the PKU-Market-PCB dataset. For the missing hole category, both models detect all defects, but SCP-DETR achieves higher confidence scores. For the mouse bite and spurious copper categories, RT-DETR exhibits missed detections, whereas SCP-DETR detects all defects while maintaining high confidence scores. For the open circuit category, neither model detects the defect in the upper-right corner. Moreover, RT-DETR incorrectly classifies the defect in the upper-left corner as a mouse bite, resulting in a false positive. For the short and spur categories, both models exhibit missed detections, but RT-DETR misses two defects, while SCP-DETR misses only one. In summary, across all categories, SCP-DETR achieves higher confidence scores for each defect compared to RT-DETR. For more challenging categories, SCP-DETR may exhibit some missed detections, but the miss rate is significantly lower than that of RT-DETR, and no false positives are observed.

## 5. Conclusion

In this paper, we conduct an in-depth analysis of the characteristics of PCB defects, which primarily involve the challenges of complex background information that is difficult to separate from defect regions and the small size of the defects. To address the aforementioned issues, we designed a unique fusion module named CO-Fusion. This module processes three input features at different scales, thereby enhancing the overall modeling capability. Additionally, to further improve the detection performance for small objects, we built upon RT-DETR-R18 and primarily modified its neck pyramid structure. As a result, we developed the SCP-DETR network, which effectively extracts small-object information for PCB defect detection.

We conducted extensive experimental analysis on the open-source PKU-Market-PCB dataset from Peking University, comparing our approach with other state-of-the-art algorithms. The results show that our proposed model improves Precision and Recall by 1.4% and 1%, respectively, increases mAP@0.5 by 3%, and boosts mAP@0.5:0.95 by 2.2%. These improvements come with only a slight increase in parameter count, indicating that our model can achieve better detection performance with nearly the same computational cost. Although the detection accuracy of the model has improved, future research needs to focus on detecting challenging samples such as spur defects. We will also continue to explore the difficult task of achieving a balance between model performance and parameter count in subsequent studies.

In summary, the SCP-DETR network model has achieved a significant breakthrough in the field of PCB defect detection. The model’s substantial performance improvements and practical application potential have been fully validated. By addressing existing limitations and exploring new research directions, we are confident in providing feasible and effective solutions for real-world engineering applications.
